# Pharmacological Disruption of Phosphorylated Eukaryotic Initiation Factor-2α/Activating Transcription Factor 4/Indian Hedgehog Protects Intervertebral Disc Degeneration via Reducing the Reactive Oxygen Species and Apoptosis of Nucleus Pulposus Cells

**DOI:** 10.3389/fcell.2021.675486

**Published:** 2021-06-07

**Authors:** Junping Bao, Zhanyang Qian, Lei Liu, Xin Hong, Hui Che, Xiaotao Wu

**Affiliations:** ^1^Spine Center, The Affiliated Zhongda Hospital of Southeast University, Nanjing, China; ^2^School of Medicine, Southeast University, Nanjing, China; ^3^Faculty of Medicine, Medical Center, Albert-Ludwigs-University of Freiburg, Freiburg im Breisgau, Germany

**Keywords:** intervertebral disc degeneration, apoptosis, reactive oxygen species, eIF2 alpha, activating transcription factor 4, Indian hedgehog, cyclopamine, ISRIB

## Abstract

Excessive reactive oxygen species (ROS) and apoptosis in nucleus pulposus (NP) cells accelerate the process of intervertebral disc degeneration (IDD). Here, we integrated pathological samples and *in vitro* and *in vivo* framework to investigate the impact of phosphorylation of eukaryotic initiation factor-2α (eIF2α)/activating transcription factor 4 (ATF4)/Indian hedgehog (Ihh) signaling in the IDD. From the specimen analysis of the IDD patients, we found phosphorylated eIF2α (p-eIF2α), ATF4 and Ihh protein levels were positively related while the NP tissue went degenerative. *In vitro*, tumor necrosis factor (TNF)-α caused the NP cell degeneration and induced a cascade of upregulation of p-eIF2α, ATF4, and Ihh. Interestingly, ATF4 could enhance Ihh expression through binding its promoter region, and silencing of ATF4 decreased Ihh and protected the NP cells from degeneration. Moreover, ISRIB inhibited the p-eIF2α, which resulted in a suppression of ATF4/Ihh, and alleviated the TNF-α-induced ROS production and apoptosis of NP cells. On the contrary, further activating p-eIF2α aggravated the NP cell degeneration, with amplification of ATF4/Ihh and a higher level of ROS and apoptosis. Additionally, applying cyclopamine (CPE) to suppress Ihh was efficient to prevent NP cell apoptosis but did not decrease the ROS level. In an instability-induced IDD model in mice, ISRIB suppressed p-eIF2α/ATF4/Ihh and prevented IDD via protecting the anti-oxidative enzymes and decreased the NP cell apoptosis. CPE prevented NP cell apoptosis but did not affect anti-oxidative enzyme expression. Taken together, p-eIF2α/ATF4/Ihh signaling involves the ROS level and apoptosis in NP cells, the pharmacological disruption of which may provide promising methods in preventing IDD.

## Introduction

Intervertebral disc degeneration (IDD) is a potential cause of chronic degenerative disc disease and pathophysiological disorders, including discogenic back pain, disc herniation, spinal stenosis, and spinal instability (Dowdell et al., 2017). The normal intervertebral disc comprises the upper and lower cartilage endplates, the peripheral fibrous annulus, and the central nucleus pulposus (NP) tissues, which absorb shocks between the vertebral bodies and play an essential role in the normal flexion, extension, and rotation of the spine (Vo et al., 2016). The exact mechanism of IDD has not been fully elucidated so far. Nevertheless, it is clear that it is the result of multiple factors, containing biomechanical factors, molecular biology factors, and genetic metabolism factors. These inducements lead to changes in the microenvironment of the intervertebral disc, resulting in the NP cells gradually losing their original phenotype and functions (Roberts et al., 2006).

Although the NP cells only account for 1–5% of the NP’s volume, the extracellular substances secreted by NP cells (such as type II collagen and aggrecan) maintain the typical hydrostatic properties of the intervertebral disc. Excessive apoptosis in NP cells directly breaks the dynamic balance between the destruction and synthesis of the extracellular matrix (ECM) (Zhang et al., 2016). The damage to the amount or quality of the NP cells causes the transformation of collagen type and the decrease in aggrecan and water content, which infers that excessive NP cell apoptosis may be the origin of the IDD (Ding et al., 2013). During IDD, there is a serious imbalance between the production of intracellular free radicals and antioxidant defenses, resulting in the accumulation of reactive oxygen species (ROS), causing abnormal cellular function, which is an important factor in apoptosis (Feng et al., 2017). ROS act as signaling messengers in a variety of apoptosis signaling pathways, such as phosphatidylinositol-3-kinase (PI3K)/Akt pathway (Song et al., 2017), nuclear factor erythroid 2-related factor 2 (Nrf-2)/heme oxygenase-1 (HO-1) pathway (Xu et al., 2020), and silent information regulator 1 (SIRT1)/endothelial nitric oxide synthase (eNOS)/nuclear factor-κB (NF-κB) pathway (Li et al., 2018). However, our understanding of the ROS-sensitive signaling network in NP cells is limited.

Activating transcription factor 4 (ATF4) is a member of the ATF/CREB family, related to amino acid and glucose metabolism, intracellular anti-oxidative stress response, and transcriptional regulation of inflammatory factors. ATF4 overexpression can trigger a cascade of ROS and induce apoptosis (Wortel et al., 2017; Zong et al., 2017). On the contrary, the disorder of ATF4 alleviates the ROS production and apoptotic process in various tissues and cell types (Fan et al., 2020). As an upstream of ATF4, the phosphorylation of eukaryotic initiation factor-2α (eIF2α) preferentially facilitates the ATF4 activation, which transduces signals to the cytoplasm and nucleus to cause the imbalance of cellular homeostasis (Harding et al., 2003; Liu and Zhang, 2020). Inhibiting the phosphorylated eIF2α (p-eIF2α) leads to a decrease in the post-translational activation of ATF4 (Zhao et al., 2020a; Zhou et al., 2020). The current literature of ATF4 on the process of apoptosis mainly mentions its positive regulation. However, some people claim that inhibiting ATF4 can also increase apoptosis. Wu et al. (2017) announced that the silencing of ATF4 led to the activation of an endoplasmic reticulum (ER) stress-specific caspase cascade and aggravated the apoptosis during chondrocyte (CH) differentiation. Interestingly, a previous study pointed out that a deficiency in the ATF4 pathway suppressed the glucose-deprivation caused autophagy and increased apoptosis in the early-stage, but weakened the autophagy and decreased ROS accumulation and apoptosis in the late-stage in NP cells (Chang et al., 2017). At present, whether ATF4 has a regulatory effect on the NP cells is not fully elucidated, and its specific mechanism is still unknown.

NP cells have a phenotype resembling CHs, and IDD is similar to osteoarthritis (OA) in etiology. Calcification is a phenomenon that occurs in the degenerated cartilage and intervertebral disc, involving hypertrophic differentiation of CHs or NP cells, which accelerates the apoptosis process (Rutges et al., 2010; Bertrand et al., 2020). The hypertrophic differentiation usually accompanies the upregulation of collagen X (Col-X), matrix metalloproteinase-13 (MMP13), and Indian hedgehog (Ihh) expression (Rutges et al., 2010; Liu et al., 2019). Ihh plays a role in the maturation, degeneration, and calcification of the intervertebral disc (Wang et al., 2015; Bach et al., 2019), and it has a potential interaction with the ATF4 gene (Wang et al., 2009; Takahata et al., 2012). However, limited literature is available regarding the crosstalk of ATF4 and Ihh in the IDD. To take a further step, our study investigated a pharmacological interruption of p-eIF2α/ATF4/Ihh in the IDD by employing the human NP cells *in vitro* and the mouse model *in vivo*. What we identified provides a new understanding and pharmacological attempt in preventing the progress of IDD.

## Materials and Methods

### Reagents and Antibodies

Collagenase XI, dispase II, tumor necrosis factor (TNF)-α, bovine serum albumin (BSA), and biotinylated IgG were from Sigma (Ohio, United States); DMEM/F12 medium, mixture of penicillin/streptomycin, and fetal bovine serum (FBS) were from Thermo (Massachusetts, United States); Masson’s Trichrome Stain Kit and Safranin O solution were from Solarbio (Shanghai, China); anti-ATF4 (ab216839 and ab184909), anti-Ihh (ab52919), anti-Col-X (ab49945, and ab260040), anti-superoxide dismutase 1 (SOD1, ab51254), anti-MMP13 (ab219620 and ab51072), anti-IgG (ab171870), anti-eIF2α (ab169528), anti-p-eIF2α (ab32157), anti-β-actin (ab8227), goat anti-mouse IgG H&L (Alexa Fluor^®^ 488) (ab150113), and goat anti-rabbit IgG H&L (Alexa Fluor 647) (ab150083) were from Abcam (Cambridge, United Kingdom); Vectastain Elite ABC reagent, Annexin V-EGFP/PI kit (KGA102), and ROS detection kit(KGAF018) were obtained from KeyGen (Nanjing, China); cyclopamine (CPE), BTdCPU (BTd), and ISRIB were from Selleck (Shanghai, China); Collagen II Polyclonal Antibody (Col-II, PA5-99159), cleaved Caspase3 antibody (PA5-114687), Lipofectamine 2000 transfection reagent, and protein isolation (89900, 78510) were from Thermo Fisher Scientific (Waltham, United States); SimpleChIP Plus Sonication Chromatin IP Kit (Magnetic Beads) was from Cell Signaling Technology (56383, Massachusetts, United States); other reagents not precisely specified were from Beyotime (Shanghai, China).

### Human Nucleus Pulposus Tissue Collection

Based on the magnetic resonance imaging (MRI), we recruited eight patients undergoing spine fracture surgery (exclusion criteria: IDD history) as the source of the healthy NP tissues and 16 patients undergoing intervertebral disc surgery as the source of the degenerated NP tissues at our hospital. They signed the informed consent and approved the publication of MRI and research results. Specimens were stored in a sterile medium and immediately transported from the hospital to the laboratory in an ice box. NP tissues were divided into three groups according to the Pfirrmann score (Urrutia et al., 2016), of which grade 1 means no visible IDD, grade 3 means mild IDD, and grade 5 means severe IDD. This study was supported by the Ethics Committee of the Affiliated Zhongda Hospital of Southeast University (registered number: 2017ZDKYSB095) and carried out following the Declaration of Helsinki.

### Nucleus Pulposus Cell Isolation and Cell Culture

We isolated human NP cells from the NP tissues (*n* = 8) according to the method previously described (Che et al., 2020). Briefly, NP tissues were cut into small pieces and digested in DMEM/F12 medium containing collagenase XI (1,500 U/ml), dispase II (2.4 U/ml), 1% penicillin/streptomycin, and 10% FBS at 37°C overnight. The NP cells were collected from the digested solution after centrifugation, and the first or second generation of NP cells was used for the experiments. To establish the NP cell degeneration model, we stimulated the NP cells with TNF-α. We blocked the ATF4 gene expression of NP cells by small interfering RNA (siRNA) transfection. Besides, we inhibited the Ihh expression by CPE, reversed the effects of eIF2α phosphorylation by ISRIB, and promoted eIF2α phosphorylation by BTd.

### Small Interfering RNA Interference

Human ATF4 siRNA (ID: 122168) and the negative control siRNA (NC-siRNA, AM4611) were purchased from Thermo Fisher Scientific (Waltham, United States) and transfected by Lipofectamine 2000 into NP cells based on the manufacturer’s instruction. The transfection efficiency was determined by western blot (WB).

### Western Blot

We isolated the total proteins from human NP tissues (*n* = 6 per group) or cultured NP cells (*n* = 7 per group) by a protein extraction kit based on the manufacturer’s instruction. Then, the protein of each sample was added in the sodium dodecyl sulfate–polyacrylamide gel electrophoresis (SDS-PAGE) and transferred onto the polyvinylidene difluoride (PVDF) membrane. After being blocked with 5% milk, the membranes were incubated with the primary antibodies against eIF2α, p-eIF2α, ATF4, Ihh, SOD1, MMP13, Caspase3, or β-actin overnight at 4°C. After 1 h secondary antibody incubation at room temperature, the membranes were visualized with enhanced chemiluminescence (ECL) substrate. The gray value of each blot was measured using ImageJ software (Media Cybernetics, Inc.). Blots of each group were examined by two researchers blinded to the experiments and then averaged for analysis.

### Chromatin Immunoprecipitation Assay

We searched the upstream 2,000 bp section of the promoter region of the Ihh gene from the National Center for Biotechnology Information database, from which we identified two putative DNA-binding sites for the ATF4 in the Ihh promoter using the JASPAR core database^1^. Chromatin immunoprecipitation (ChIP) assay was used to pull down the protein with ATF4 or IgG antibody. The putative DNA-binding sites of ATF4 to Ihh were verified by PCR, followed by digital imaging of agarose gel electrophoresis (AGE). PCR primers for PCR were designed by Primer Premier (Supplementary Table 1).

### Plasmid Constructs and Luciferase Reporter Gene Assay

We used pGL4.23-basic luciferase plasmid to load the wild-type (WT) or mutant Ihh promoter segments. Renilla plasmid (pRL) was used as a fluorescent internal reference. Additionally, the ATF4 sequence vector was used to increase the ATF4 expression. For negative control, empty vector and pGL4.23 were also used in the co-transfection with Lipofectamine 2000 based on the manufacturer’s instruction. Finally, the luciferase activity was normalized to Renilla luciferase activity. All the vectors and plasmids were synthesized by the Promoterbio Lab (Taizhou, China).

### Dual Immunofluorescence

We determined the Col-X and Col-II protein expression of NP cells with a dual immunofluorescence (IF) method. After treatments, NP cells (*n* = 7 per group) were washed three times using phosphate-buffered saline (PBS), fixed with 4% paraformaldehyde (PFA), incubated in 0.01% Triton X-100 for 15 min separately, and blocked with 5% BSA for 1 h at room temperature. NP cells were incubated with primary antibodies: mouse monoclonal anti-Col-X and rabbit polyclonal anti-Col-II at 4°C overnight. The next day, NP cells were washed and incubated with Alexa Fluor647-conjugated goat anti-rabbit IgG, Alexa Fluor488-conjugated goat anti-mouse IgG, and DAPI for 45 min in the dark at room temperature. The staining intensity was visualized using a fluorescence microscope (Leica, Wetzlar, Germany) and analyzed by ImageJ. Cells of each group were examined by two researchers blinded to the experiments and then averaged for analysis.

### Mouse Intervertebral Disc Degeneration Model by Surgically Induced Instability

Twenty-five female and 25 male C57 background BL/6J mice (8 weeks, 18–22 g; purchased from the model animal research center of Nanjing University) were randomly divided into five groups (*n* = 10 per group). We established the IDD model according to the previous description (Oichi et al., 2018). Briefly, the mice were generally narcotized with 2–3% isoflurane. We exposed the bilateral facet joints of the L3–L4 level and transected the bilateral inferior articular processes and supra- and interspinous ligaments by microscissors (Supplementary Figure 1). After the operative fields were closed, the mice were put back to the cages and injected with normal saline (NS) or ISRIB/CPE subcutaneously once a day for 8 weeks (inclusion criteria: successful surgery). In the control group, mice were sutured after the incision of skin and muscle and did not undergo lumbar surgery to induce instability. In the NS group, mice underwent lumbar surgery and injected with NS. In the ISRIB group, mice were injected with 0.25 mg/kg (Sidrauski et al., 2013) of ISRIB after lumbar surgery. In the CPE group, mice were injected with 50 mg/kg (Berman et al., 2003) of CPE after lumbar surgery. In the ISRIB + CPE group, we injected the mice with 0.25 mg/kg of ISRIB and 50 mg/kg of CPE following 1 h interval time. Injections were started 3 days after surgery, and the mice were sacrificed after 8 weeks’ treatment. The cages were covered with sawdust with a 12 h light/12 h dark cycle and a constant temperature of 20°C. All of the mice were free to access food and water. The animal use was registered in the animal experimental ethical inspection form of Southeast University (20190913003).

### X-Ray Study

After the mice were sacrificed, we separated the lumbar spines (*n* = 6 per group, from L1 to L6) and took X-ray images (DRE Stationary DR Digital X-Ray System, United States) to measure the disc height and vertebral body. We determined the disc height of the L3–L4 level by the calculation of the intervertebral disc height index (DHI): 2 × (height of the posterior + middle + anterior parts of the disc)/(height of the posterior + middle + anterior parts of the two adjacent vertebral body) (Supplementary Figure 2). Mice of each group were examined by two researchers blinded to the experiments and then averaged for analysis.

### Masson’s and Safranin O Staining

After fixation in 4% PFA solution for 24 h, the mouse lumbar spines (*n* = 6 per group, L3–L4) were decalcified with 10% ethylenediaminetetraacetic acid for 7 days. To prepare the histological staining, both human NP tissues and mouse spines were embedded in paraffin and sectioned into 5 μm-thick slices. Then, the slices underwent deparaffinization and hydrate with gradient alcohol. The fibrosis progress of NP tissues was visualized with Masson’s stain (Nagatoya et al., 2002), and the proteoglycan (PG) content was visualized with Safranin O stain (Kiviranta et al., 1985). The slices were imaged using an Axio Imager 2 microscope and Zen2TM software (Carl Zeiss, Germany). The details of the staining protocol were based on the manufacturer’s instructions. All sections were examined by two researchers blinded to the experiments and then averaged for analysis.

### Flow Cytometry Analyses of Apoptosis and Reactive Oxygen Species

After treatments, NP single-cell suspensions (*n* = 7 per group) were collected in PBS. The apoptotic population of NP cells was determined using Annexin V-EGFP/PI kit based on the manufacturer’s instructions. The total cell apoptosis was determined by the sum of early and late apoptotic cells. For the measurement of ROS production, cells were incubated with 10 μM of DCFH-DA for 1 h at 37°C in dark, and then the medium was changed to DMEM/F12 for 30 min. Subsequently, ROS production was detected at a wavelength of 488 nm.

### Immunohistochemistry

After fixation in 4% PFA solution for 24 h, both human NP tissues and decalcified mouse spines (*n* = 6 per group) were embedded in paraffin and sectioned into 5 μm-thick slices. After the samples were deparaffinized and hydrated with gradient alcohol, we performed the immunohistochemistry (IHC) as a previous description (Yukata et al., 2018). After antigen retrieval and endogenous peroxidase inactivation, the slices were blocked with 10% goat serum and incubated with primary antibodies against Col-II, Col-X, eIF2α, p-eIF2α, ATF4, Ihh, SOD1, MMP13, and Caspase3 at 4°C overnight. After washing, we treated the slices with biotinylated IgG, Elite ABC reagent, and 3,3-diaminobenzidine in order. Finally, the slices were counterstained by hematoxylin. The slices were imaged using an Axio Imager 2 microscope and Zen2TM software (Carl Zeiss, Germany). The positive cell rate indicated the ratio of the number of positive nuclei and/or cytoplasm to the number of all hematoxylin-labeled cells. All sections were examined by two researchers blinded to the experiments and then averaged for analysis.

### Statistical Analysis

Analyses were performed by SPSS (version 22), and data were expressed as mean ± standard deviation (*SD*). One-way ANOVA with Sidak’s multiple comparison test was used to determine the differences between the groups. Two-sided *P* < 0.05 showed statistical significance between groups. Cartograms were generated from GraphPad Prism (version 8).

## Results

### Phosphorylated Eukaryotic Initiation Factor-2α/Activating Transcription Factor 4/Indian Hedgehog Increases in the Degenerated Nucleus Pulposus Tissue

**FIGURE 1:**
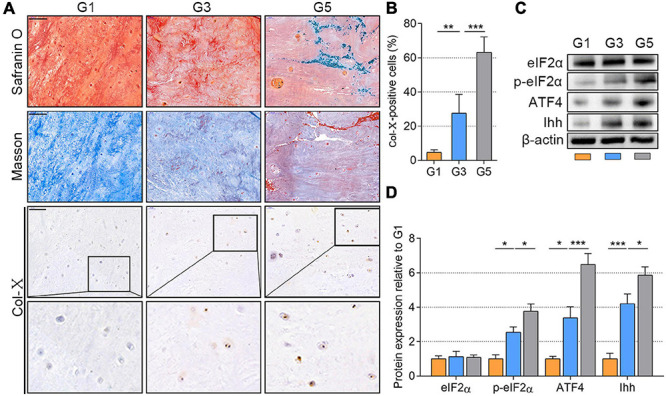
p-eIF2α, ATF4, and Ihh expression in human NP tissues with different degeneration degrees (G1, G3, and G5 according to Pfirrmann grade). **(A)** Representative images of Safranin O staining with collagen and NP cells appearing orange and fibers blue/violet; Masson’s staining with collagen and NP cells appearing blue and fibers red; IHC staining for Col-X (scale bar = 100 μm) and **(B)** quantification of Col-X-positive cells (%). **(C)** eIF2α, p-eIF2α, ATF4, and Ihh protein levels were assessed by WB, **(D)** measured by densitometric analyses, and expressed as folds relative to G1 samples. Data are presented as means ± *SD* (*n* = 6). *p < 0.05, **p < 0.01; ***p < 0.001; the orange column indicates G1, the blue column indicates G3, and the gray column indicates G5. p-eIF2α, phosphorylated eukaryotic initiation factor-2α; ATF4, activating transcription factor 4; Ihh, Indian hedgehog; NP, nucleus pulposus; IHC, Immunohistochemistry; Col-X, collagen X; eIF2α, eukaryotic initiation factor-2α; WB, western blot.

Based on the Pfirrmann classification method (Urrutia et al., 2016), we collected the NP tissues of grades 1, 3, and 5 (G1, G3, and G5, respectively) from the spine fracture patients and IDD patients who needed surgery (patients’ information is available in Supplementary Table 2). NP tissues of G1 indicate a nearly healthy condition without visible IDD, those of G3 indicate mild IDD, and those of G5 indicate severe IDD (representative MRI of each grade shown in Supplementary Figure 3). To value the degenerated status of NP tissues, we analyzed the PG content using Safranin O stain (Kiviranta et al., 1985) and the fibrosis with Masson’s stain (Nagatoya et al., 2002). As shown in Figure 1A, the orange area of Safranin O presented the PGs, which was gradually discolored from G1 to G5 and replaced by the fiber (blue color). Besides, the collagen content (blue color) of NP tissues was also gradually occupied by the fiber (orange color). Meanwhile, the Col-X-positive cells were also significantly increased from the G1 to G5 group (*P* < 0.01; Figures 1A,B). Additionally, the results of WB analysis indicated that the total eIF2α was stable and p-eIF2α protein gradually accumulated as the NP tissues degenerated from G1 to G5, which was accompanied by an upregulation of ATF4 and Ihh expression (*P* < 0.05; Figures 1C,D). Therefore, p-eIF2α, ATF4, and Ihh were simultaneously increased as the IDD worsened.

### Activating Transcription Factor 4 Deficiency Alleviates the Tumor Necrosis Factor-α-Induced Indian Hedgehog Upregulation

**FIGURE 2:**
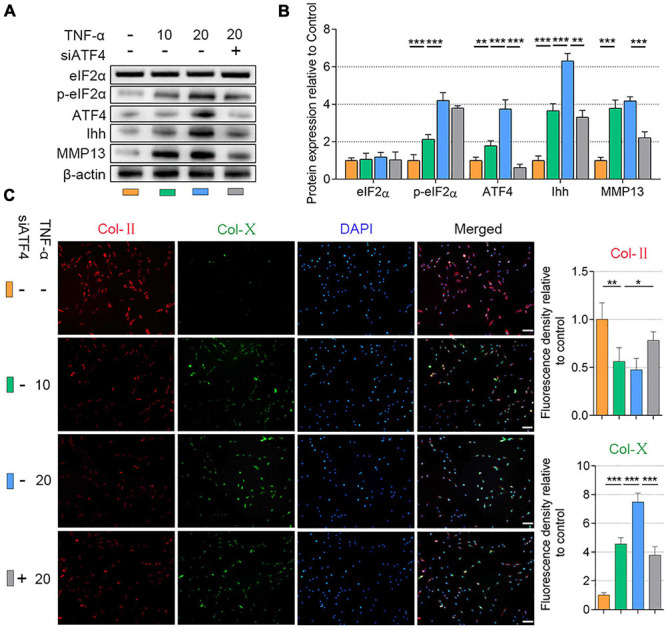
Silencing of ATF4 suppressed Ihh expression and human NP cell degeneration. NP cells were treated with low dose (10 ng/ml) or high dose (20 ng/ml) of TNF-α for 3 days to induce degeneration and transfected with siATF4 to silence the ATF4 gene expression. **(A)** eIF2α, p-eIF2α, ATF4, Ihh, and MMP13 protein levels were assessed by WB, **(B)** measured by densitometric analyses, and expressed as folds relative to control. **(C)** Representative images of IF staining with Col-II and Col-X and measured by densitometric analyses and expressed as folds relative to control (scale bar = 100 μm). Data are presented as means ± *SD* (*n* = 7). *p < 0.05; **p < 0.01; ***p < 0.001; the orange column indicates control NP cells without any treatments, the green column indicates NP cells treated with 10 ng/ml of TNF-α, the blue column indicates NP cells treated with 20 ng/ml of TNF-α, and the gray column indicates siATF4 transfected NP cells treated with 20 ng/ml of TNF-α. ATF4, activating transcription factor 4; Ihh, Indian hedgehog; NP, nucleus pulposus; TNF, tumor necrosis factor; p-eIF2α, phosphorylated eukaryotic initiation factor-2α; eIF2α, eukaryotic initiation factor-2α; MMP13, matrix metalloproteinase-13; WB, western blot; IF, immunofluorescence; Col-II, collagen II; Col-X, collagen X.

We established NP cell degeneration model using the stimulation of TNF-α (Zhao et al., 2020b). To elucidate whether the ATF4 affects p-eIF2α and Ihh expression in NP cells, the ATF4 gene-silenced NP cells were applied (the NC-siRNA is shown in Supplementary Figure 4). The NP cells (at the density of 1.5 × 10^4^ per well) were cultured in 12-well plates and treated with TNF-α (10 or 20 ng/ml) for 3 days. Compared with the control, the protein expression of p-eIF2α, ATF4, and Ihh was increased after the stimulation of TNF-α in a dose-dependent manner. However, eIF2α protein was not changed after TNF-α treatment. MMP13 was increased in 10 ng/ml of TNF-α stimulation but not changed by increasing dose. In the same stimulation of 20 ng/ml of TNF-α, silencing the ATF4 gene significantly reduced the Ihh and MMP13 protein expression but did not affect the p-eIF2α protein expression (*P* < 0.01; Figures 2A,B). Additionally, TNF-α treatment successfully reduced the Col-II and promoted Col-X expression in NP cells, which was alleviated after the ATF4 gene was deficient (*P* < 0.05; Figure 2C). Thus, the suppression of ATF4 led to the prevention of Ihh expression and a weakened degeneration of NP cells.

### Activating Transcription Factor 4 Enhances Indian Hedgehog Expression Through Binding to Its Promoter

**FIGURE 3:**
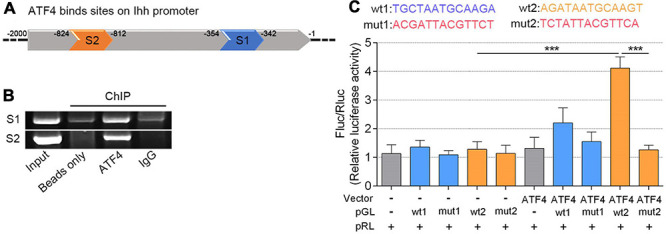
ATF4 bound the Ihh gene promoter and promoted its expression of human NP cells. **(A)** Two predicted sites (S1 and S2) that ATF4 bound on the promoter region of Ihh. **(B)** AGE indicated two putative DNA sequences of Ihh immunoprecipitated by the anti-ATF4-coded beads. **(C)** Dual-luciferase reporter assay in NP cells co-transfected with the reporter plasmid and ATF4. Structural schematic of the wt and mutant sequences of S1 and S2 in the human Ihh promoter region is shown. Luciferase activity driven by the wt2 Ihh promoter was more pronounced following ATF4 treatment, and it was not driven by the mut2 luciferase reporter upon ATF4 treatment. In contrast, luciferase activity driven by the wt2 luciferase reporter decreased in the absence of ATF4. Luciferase activity showed no difference when driven by wt1 luciferase reporter. Data are presented as means ± *SD* (*n* = 3). ***p < 0.001, - indicates not applied, and + indicates applied. ATF4, activating transcription factor 4; Ihh, Indian hedgehog; AGE, agarose gel electrophoresis; wt, wild type.

As a lesser level of ATF4 results in a less Ihh expression, we speculated that a higher level of ATF4 would trigger more Ihh production. Based on a previous study from Wang et al. (2009), we attempted to confirm that ATF4 promotes the Ihh expression by activating its promoter region. Fortunately, we found two putative DNA-binding sites for the ATF4 in the upstream 2,000 bp section of the promoter region of the Ihh gene (schematic in Figure 3A; details in Supplementary Figure 5; Bryne et al., 2008). In the ChIP assessment, we immunoprecipitated the ATF4 protein in the chromatin lysates of NP cells using the anti-ATF4-coded beads and successfully amplificated these two putative DNA sequences of Ihh by PCR (lane 3 of Figure 3B), indicating that the ATF4 protein indeed bound to the putative promoter region of the Ihh gene. In contrast, the beads coding nothing or IgG as negative control did not carry the putative DNA sequences of Ihh (lanes 2 and 4 of Figure 3B). The whole chromatin fragment of NP cells was used as a positive control to verify the specific PCR primers for DNA amplification (input, lane 1 of Figure 3B).

We further performed a dual-luciferase reporter gene assay to test whether ATF4 protein is efficient to induce Ihh expression via the two putative binding sites (wt1 and wt2). We transfected NP cells with ATF4-coding Vector to upregulate the ATF4 protein level. Meanwhile, the pGL luciferase reporter carrying the binding promoter sequence (wt1 or wt2) or mutant sequence (mut1 or mut2) was also used to mark the Ihh promoter. The empty vector and pGL luciferase reporter were used as a negative control. Additionally, we used the pRL-Renilla Luciferase Vector to normalize the pGL luciferase of each group. The wt and mut sequences involved in the pGL are shown in Figure 3C. Interestingly, we found that ATF4 overexpression could significantly increase the pGL-wt2 luciferase activity, which was abolished in the transfection of pGL-mut2. The luciferase activity of pGL-wt2 in NP cells was not significantly increased when the ATF4 vector was not transfected. In addition, we did not find the same change that overexpression of ATF4 could induce the pGL-wt1 luciferase expression (*P* < 0.001; Figure 3C). Therefore, we concluded that ATF4 could activate the Ihh promoter via the sequence of wt2, and the increased Ihh expression during the NP cell degeneration could be regulated by ATF4.

### Eukaryotic Initiation Factor-2α Phosphorylation Promotes Activating Transcription Factor 4/Indian Hedgehog Expression, Reactive Oxygen Species Accumulation, and Apoptosis

**FIGURE 4:**
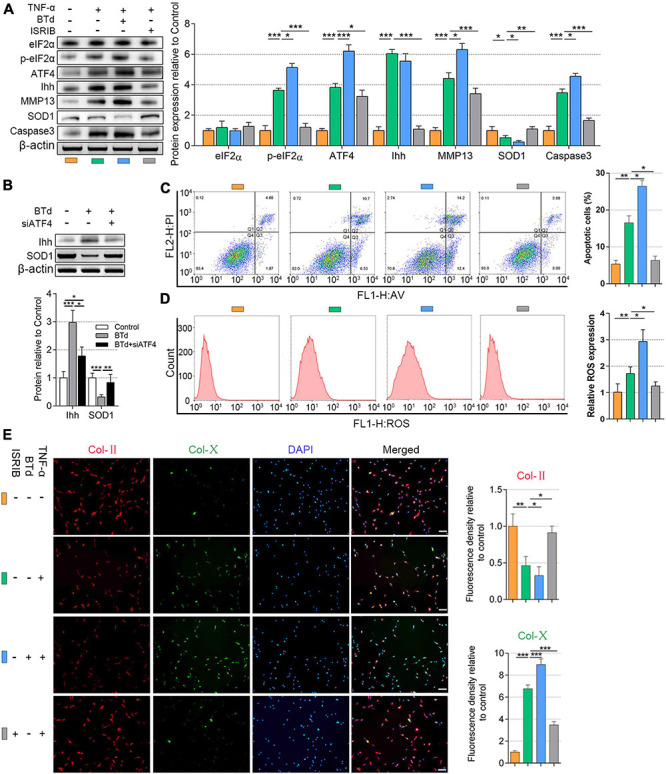
Phosphorylation of eIF2α affected ATF4, Ihh expression, oxidative stress, and apoptosis of human NP cells. NP cells were treated with 20 ng/ml of TNF-α for 3 days or simultaneously applied with BTd and ISRIB. **(A)** eIF2α, p-eIF2α, ATF4, Ihh, MMP13, SOD1, and Caspase3 protein levels were assessed by WB, measured by densitometric analyses, and expressed as folds relative to control. **(B)** Normal NP cells or ATF4-silenced NP cells were treated with BTd, and the Ihh expression and SOD1 expression were tested by WB. Flow cytometry assay and the corresponding statistical analysis of **(C)** cell apoptosis (%) and **(D)** ROS expression. **(E)** Representative images of IF staining with Col-II and Col-X, measured by densitometric analyses and expressed as folds relative to control (scale bar = 100 μm). Data are presented as means ± *SD* (*n* = 7). *p < 0.05; **p < 0.01; ***p < 0.001; the orange column indicates control NP cells without any treatments, the green column indicates NP cells treated with TNF-α, the blue column indicates NP cells treated with TNF-α and BTd, and the gray column indicates NP cells treated with TNF-α and ISRIB. eIF2α, eukaryotic initiation factor-2α; ATF4, activating transcription factor 4; Ihh, Indian hedgehog; NP, nucleus pulposus; TNF, tumor necrosis factor; p-eIF2α, phosphorylated eukaryotic initiation factor-2α; MMP13, matrix metalloproteinase-13; SOD1, superoxide dismutase 1; WB, western blot; ROS, reactive oxygen species; IF, immunofluorescence; Col-II, collagen II; Col-X, collagen X.

Recent studies have reported that eIF2α phosphorylation plays a role in the transcription of ATF4 (B’Chir et al., 2013; Feng et al., 2019), which has not been verified in the NP cells. To further understand the upstream of mediation of ATF4, we upregulated the p-eIF2α superimposing on the TNF-α-induced NP cell degeneration. NP cells (1.5 × 10^4^ per well in 12-well plates) were basically cultured with 20 ng/ml of TNF-α to induce degeneration. The BTd (10 μM) was extra applied to aggravate the phosphorylation of eIF2α, and ISRIB (5 nM) was used for preventing the phosphorylation of eIF2α. All the cells were cultured for 3 days. From the protein patterns, the expression of p-eIF2α, ATF4, Ihh, MMP13, and Caspase3 was increased within TNF-α treatments compared with the control. Meanwhile, SOD1 was decreased, and total eIF2α protein content did not significantly change. Except for Ihh, BTd supplement aggravated the phosphorylation of eIF2α, which led to a further increase of ATF4, MMP13, and Caspase3 and a further decrease of SOD1 compared with the TNF-α group. On the contrary, ISRIB suppressed the p-eIF2α level; reduced the ATF4, Ihh, MMP13, and Caspase3 protein expressions; and increased the SOD1 protein expression as compared with the TNF-α group (*P* < 0.05; Figure 4A). To clarify the direct effect of phosphorylation of eIF2α on Ihh and SOD1, we treated the NP cells with BTd (10 μM) for 3 days. The Ihh protein level was significantly increased, and SOD1 was decreased. After ATF4 was silenced, the Ihh expression was reduced but still higher than that of the control group, and the SOD1 expression was almost rescued (*P* < 0.05; Figure 4B). According to the results of flow cytometry, an increase of p-eIF2α aggravated the apoptosis and ROS content, and suppression of p-eIF2α alleviated the apoptosis and ROS content in NP cells (*P* < 0.05; Figures 4C,D). Moreover, the BTd supplement accelerated the loss of Col-II and the production of Col-X. When the p-eIF2α was suppressed by ISRIB, the Col-II protein expression was protected, and Col-X was inhibited (*P* < 0.05; Figure 4E).

### Suppression of Indian Hedgehog Alleviates the Tumor Necrosis Factor-α-Induced Hypertrophy and Apoptosis

**FIGURE 5:**
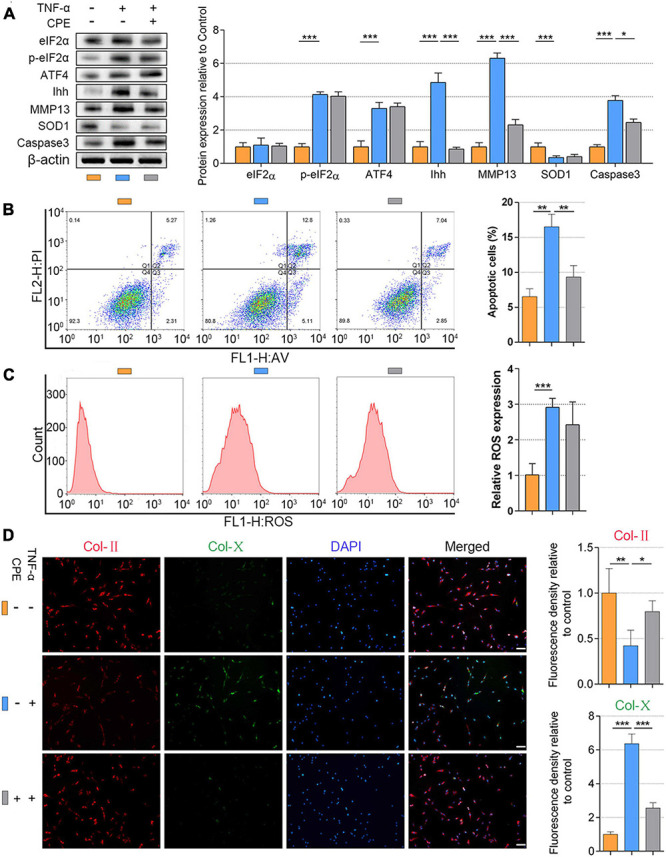
Inhibiting Ihh expression decreased hypertrophy and apoptosis in human NP cells. NP cells were treated with 20 ng/ml of TNF-α for 3 days or simultaneously applied CPE. **(A)** eIF2α, p-eIF2α, ATF4, Ihh, MMP13, SOD1, and Caspase3 protein levels were assessed by WB, measured by densitometric analyses, and expressed as folds relative to control. Flow cytometry assay and the corresponding statistical analysis of **(B)** cell apoptosis (%) and **(C)** ROS expression. **(D)** Representative images of IF staining with Col-II and Col-X, measured by densitometric analyses and expressed as folds relative to control (scale bar = 100 μm). Data are presented as means ± *SD* (*n* = 7). *p < 0.05; **p < 0.01; ***p < 0.001; the orange column indicates control NP cells without any treatments, the blue column indicates NP cells treated with TNF-α, and the gray column indicates NP cells treated with TNF-α and CPE. Ihh, Indian hedgehog; NP, nucleus pulposus; TNF, tumor necrosis factor; CPE, cyclopamine; eIF2α, eukaryotic initiation factor-2α; p-eIF2a, phosphorylated eukaryotic initiation factor-2a; ATF4, activating transcription factor 4; MMP13, matrix metalloproteinase-13; SOD1, superoxide dismutase 1; WB, western blot; ROS, reactive oxygen species; IF, immunofluorescence; Col-II, collagen II; Col-X, collagen X.

What we have verified was that a high level of p-eIF2α promoted the upregulation of ATF4 and triggered Ihh activation. On the contrary, suppression of eIF2α phosphorylation or silencing ATF4 would inhibit Ihh expression. The p-eIF2α/ATF4/Ihh cascade is associated with the ROS accumulation and apoptosis of NP cells. However, whether the destruction of Ihh also contributes to preventing the ROS level and apoptosis of NP cells remains unknown. Thus, we used the inhibitor of Ihh superimposing on the TNF-α stimulation. NP cells (1.5 × 10^4^ per well in 12-well plates) were treated with 20 ng/ml of TNF-α without or with the presence of CPE (50 nM) for 3 days. As described before, TNF-α significantly upregulated the protein level of p-eIF2α, ATF4, Ihh, MMP13, and Caspase3 and reduced the SOD1 level in contrast to the control. Adding CPE did not affect the eIF2α, p-eIF2α, ATF4, and SOD1 protein levels but suppressed the Ihh, MMP13, and Caspase3 protein expressions (*P* < 0.05; Figure 5A). The flow cytometry results showed the CPE suppressed the apoptosis but not ROS content within TNF-α treatment (*P* < 0.01; Figures 5B,C). Besides, the suppression of Ihh by CPE also protected Col-II expression and the inhibited Col-X expression compared with the TNF-α group (*P* < 0.05; Figure 5D).

### ISRIB/Cyclopamine Treatment Alleviates Intervertebral Disc Degeneration Induced by Instability

**FIGURE 6:**
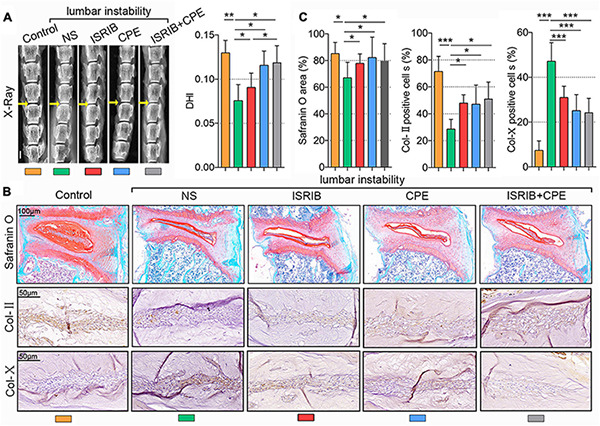
ISRIB and CPE treatment exerted a protective effect on NP cells *in vivo*. The bilateral inferior articular processes and supra- and interspinous ligaments were transected to induce lumbar instability of mice. Next, the mice were subcutaneously injected with NS, ISRIB, CPE, or the combination with ISRIB and CPE once a day after surgery and sacrificed after 8 weeks of injection. **(A)** X-ray of the mice lumbar vertebrae, and DHI of the L3–L4 level (scale bar = 2 mm). **(B)** Representative micrographs of slices stained with Safranin O (scale bar = 100 μm), Col-II, and Col-X (scale bar = 50 μm), and **(C)** quantitation of percentage of total positive area or cells (%). Data are presented as means ± *SD* (*n* = 6). *p < 0.05; **p < 0.01; ***p < 0.001; the orange column indicates control mice without lumbar instability surgery, the green column indicates mice injected with NS after lumbar instability surgery, the red column indicates mice injected with ISRIB after lumbar instability surgery, the blue column indicates mice injected with CPE after surgery, and the gray column indicates mice injected with ISRIB and CPE after lumbar instability surgery. CPE, cyclopamine; NP, nucleus pulposus; NS, normal saline; DHI, disc height index; Col-II, collagen II; Col-X, collagen X.

To further test the effects of ISRIB and CPE treatments on IDD *in vivo*, we used a mouse IDD model by surgically inducing lumbar instability (Oichi et al., 2018). As we all know, the disc height is decreased during IDD resulting from the loss of ECM and water. To measure the disc height after surgery, we took the X-ray images of the mice spine and calculated the DHI of the disc. As shown in Figure 6A, the yellow arrows indicated the surgical section (L3/4 section of the lumbar spine). Compared with the control, DHI was significantly decreased 8 weeks after surgery with an injection of NS, which was statistically rescued with the infusion of 0.25 mg/kg of ISRIB or 50 mg/kg of CPE. However, the drug combination was also efficient to protect the disc height, which was better than ISRIB but not better than CPE. The NP tissues in the mouse are too small to separate from the spine, so we measured the PG content with Safranin O staining and other protein expression using IHC methods. Among the multiple comparisons, PGs and Col-II content were reduced after the surgery, which were significantly prevented by ISRIB, CPE, or the combination treatment. Additionally, Col-X expression was upregulated after the surgery, which was alleviated by the supplement of ISRIB, CPE, and combination treatment (*P* < 0.05; Figures 6B,C).

**FIGURE 7:**
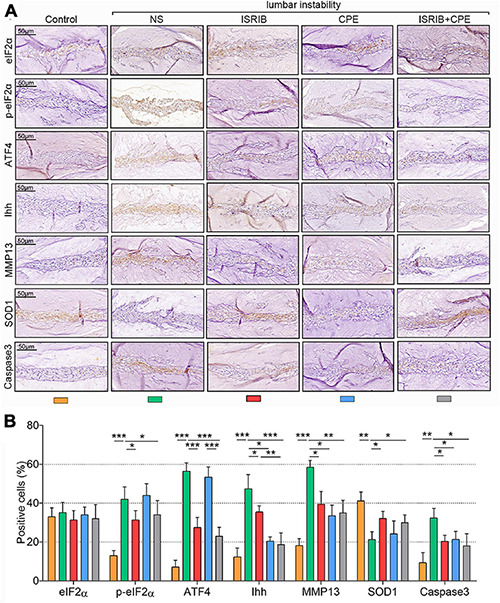
ISRIB and CPE treatment exerted anti-hypertrophy and anti-apoptosis effects of NP cells *in vivo*. The mice were treated with the procedure described above. **(A)** Representative micrographs of slices stained with eIF2α, p-eIF2α, ATF4, Ihh, MMP13, SOD1, and Caspase3. **(B)** Quantitation of percentage of total positive cells (%) (scale bar = 50 μm). Data are presented as means ± *SD* (*n* = 6). *p < 0.05; **p < 0.01; ***p < 0.001; the orange column indicates control mice without lumbar surgery to induce instability, the green column indicates mice injected with NS after lumbar surgery, the red column indicates mice injected with ISRIB after lumbar surgery, the blue column indicates mice injected with CPE after lumbar surgery, and the gray column indicates mice injected with ISRIB and CPE after lumbar surgery. CPE, cyclopamine; NP, nucleus pulposus; eIF2α, eukaryotic initiation factor-2α; p-eIF2α, phosphorylated eukaryotic initiation factor-2α; ATF4, activating transcription factor 4; Ihh, Indian hedgehog; MMP13, matrix metalloproteinase-13; SOD1, superoxide dismutase 1.

Moreover, surgery increased the p-eIF2α, ATF4, Ihh, MMP13, and Caspase3 expressions but suppressed the SOD1 expression in the NP cells. ISRIB injection alleviated the upregulation of p-eIF2α, ATF4, Ihh, MMP13, and Caspase3 expressions and prevented the reduction of SOD1 expression. In contrast, CPE did not affect the p-eIF2α, ATF4, and SOD1 levels but inhibited Ihh, MMP13, and Caspase3 expression. Even though the combined injection of ISRIB and CPE could prevent the mouse IDD (effect on MMP13, Caspase3, and SOD1), it was also not better than the ISRIB or CPE treatment alone (*P* < 0.05; Figures 7A,B). Interestingly, the total eIF2α expression was still constant after the surgery or drugs treatments.

## Discussion

In the present study, we elucidated the involvement of p-eIF2α, ATF4, and Ihh in the development of NP cell degeneration and found that disrupting the cascade of p-eIF2α/ATF4/Ihh using pharmacological methods is a promising treatment for IDD. In the patients’ NP tissue samples, the content of PGs was gradually decreased from G1 to G5 with an increase of fiber and Col-X expression. The normal anabolism and catabolism of ECM are in dynamic balance, the disorder of which will change the ECM components, thereby inducing progressive IDD (Long et al., 2019). The center layer of the intervertebral discs is mainly CH-like NP cells predominantly expressing Col-II and PGs, which helps to maintain the water content and buffer the pressure of spine (Sampara et al., 2018). During the process of IDD, NP cells gradually lose their original phenotype, and the synthesis of Col-X indicates the process of calcification and hypertrophic differentiation (Bach et al., 2019). Meanwhile, the p-eIF2α, ATF4, and Ihh levels were increased when the IDD became severe, indicating that these three genes might participate in the pathology of IDD. As a pleiotropic pro-inflammatory factor and a driving factor for IDD, TNF-α is expressed higher in degenerative NP tissues than in healthy tissues. TNF-α amplifies the inflammatory response, induces apoptosis of NP cells and degradation of ECM, and finally leads to IDD (Johnson et al., 2015). In this study, we used TNF-α to intervene in NP cells *in vitro* to stimulate the inflammatory microenvironment during IDD. TNF-α stimulation increased the phosphorylation level of eIF2α and upregulated the expression of ATF and Ihh. At the same time, the hypertrophic markers MMP13 and Col-X expression also increased significantly. When the ATF4 gene was silenced, the Ihh expression and hypertrophy-related genes upregulated by TNF-α were partly alleviated. Therefore, inhibiting ATF4 would prevent the progress of NP cell degeneration. The regulation of ATF4 by p-eIF2α has been widely reported (Kilberg et al., 2009). Whether the upregulation of ATF4 is responsible for the Ihh overexpression during IDD is still unknown.

Recent studies have announced that ATF4 has a relation with Ihh in osteoblast, CHs, and endochondral ossification (Wang et al., 2009, 2012). CHs are terminally differentiated cells, which undergo the stage of proliferation, hypertrophy, and apoptosis in OA. Col-X synthesized by hypertrophic CHs induces hydroxyapatite crystal formation and causes cartilage matrix calcification. Ihh signaling pathway plays an essential role in maintaining cartilage phenotype stability and normal articular CH metabolism (Kurio et al., 2018). Wei et al. (2012) found that Ihh overexpression is involved in CH hypertrophy and accelerates the progression of OA, and treating CHs with the inhibitor of Ihh can decrease the transcription levels of MMP13 and Col-X. During the ossification of mouse CHs, ATF4 is confirmed to transcriptionally regulate Ihh expression by binding to its promoter (Wang et al., 2009). We speculated that ATF4 could also enhance Ihh expression through the promoter manner in human NP cells. As expected, evidence from the ChIP assay indicated that ATF4 bound to two promoter regions of Ihh, and one of them can be sufficiently activated by ATF4, which explained why ATF4 deficiency also resulted in the downregulation of Ihh in NP cells.

To further clarify whether the phosphorylation of eIF2α directly triggers the upregulation of Ihh or through the mediation of ATF4, we used BTd to increase the p-eIF2α level in both original and ATF4-silenced NP cells. We noticed that the supplement of BTd did not further increase the Ihh expression within the TNF-α stimulation. We speculated that perhaps TNF-α already promoted enough Ihh expression, so continued use of BTd did not make a visible difference. Thus, we treated NP cells with BTd alone and found it was effective to upregulate Ihh expression. Interestingly, the absence of ATF4 partially but not fully counteracted the BTd that caused Ihh upregulation, which suggested that the p-eIF2α could also directly enhance the expression of Ihh. Additionally, ISRIB suppressed the phosphorylation of eIF2α and resulted in the reduction of ATF4 and Ihh. Here, the signaling cascade of p-eIF2α/ATF4/Ihh is clear, and we further explored the molecular mechanism underlying the improvement of NP cell degeneration. Oxidative stress and apoptosis play a vital role in the development of IDD (Yang et al., 2020), which are both the cues associated with p-eIF2α/ATF4 signaling. ATF4 promotes the induction of ER stress (ERS)-induced apoptosis, and its activation is intrinsically associated with the phosphorylation of eIF2α involving many stress signaling pathways (Wek et al., 2006). Excessive ERS can activate Caspase12 precursors on the ER, further activate Caspase3, and perform an apoptotic response (Zhao et al., 2010; Pang et al., 2020). In our study, TNF-α increased the Caspase3 expression and apoptosis, which was aggravated by BTd supplement. After the ISRIB is applied to interfere in the eIF2α phosphorylation, the Caspase3 expression and apoptosis were inhibited. Meanwhile, the activation of p-eIF2α inhibited the anti-oxidative enzyme SOD1 expression and promoted ROS accumulation, which was alleviated by the ISRIB supplement. The silencing of ATF4 rescued the p-eIF2α caused SOD1 suppression, indicating the mediating role of ATF4 between p-eIF2α and SOD1, which was consistent with the results of a previous study (Matus et al., 2013). Oxidative stress is a severe imbalance between the excessive production of ROS and the corresponding antioxidant defense system (Bhattacharyya et al., 2014). Therefore, the pharmacological intervention of p-eIF2α can improve the NP cell degeneration via balancing the oxidative stress and suppressing apoptosis. Compared with the many pieces of literature that have reported the role of p-eIF2α/ATF4 in oxidative stress and apoptosis, we know little about the role of Ihh in this regard.

We sought to explore whether directly inhibiting Ihh expression would rescue the NP cells from the TNF-α stimulation. Without affecting p-eIF2α and ATF4 expression, CPE significantly decreased the Ihh, MMP13, and Caspase3. However, CPE did not improve the SOD1 protein expression compared with the TNF-α group. The flow cytometry analysis also proved that inhibiting Ihh could suppress apoptosis but not decrease the ROS level. Even so, inhibition of Ihh still protected the Col-II and suppressed the MMP13 and Col-X in NP cells, indicating that CPE was useful to protect NP cell degeneration. These results reveal that Ihh participates in the regulation of p-eIF2α/ATF4 in the hypertrophy and apoptosis of NP cells, but the antioxidant effect needs to be further proven. Nowadays, CPE has been used as a promising therapeutic strategy for certain cancers and diseases associated with Ihh signaling, such as pancreatic cancer, basal cell carcinoma, and medulloblastoma (Heretsch et al., 2010). Our study, for the first time, provided a possibility that CPE might be a novel drug in the prevention of NP cell degeneration.

For *in vivo* verification, we adopted the instability-induced lumbar IDD model of the mouse to test the effects of ISRIB and CPE in the antagonism with IDD. The injection of ISRIB protected the height, PGs, and Col-II content of the disc after surgery. *In vitro*, it presented an excellent performance in suppressing the p-eIF2α, ATF4, Ihh, Col-X, and MMP13 and rescued the SOD1 expression. Additionally, CPE intervention also maintained the disc height, PGs, and Col-II expression and alleviated the Ihh, MMP13, and Caspase3 expression, though the p-eIF2α, ATF4, and SOD1 expression were not obviously affected. In these parallel experiments performed *in vivo*, pharmacological inhibition of p-eIF2α still presented an effective ability in protecting the anti-oxidative enzymes and suppressing apoptosis, which contributes to delaying disc degeneration. However, suppressing Ihh only decreased the apoptosis and did not protect the SOD1 expression. To further test whether the combination of ISRIB and CPE would magnify the effects compared with using it alone, we injected the mice with ISRIB and CPE after surgery but did not find an amplification result. Since both ISRIB and CPE can inhibit the Ihh pathway, CPE may be already sufficient to inhibit Ihh without the assistance of ISRIB. As a limitation, the analysis of the combination of ISRIB and CPE was insufficient, and more dimensional testing of the time and dose is essential to make a better understanding of their usage. The p-eIF2α/ATF4 signaling is closely related to ERS-related apoptosis from the existing reports, but the ERS-related genes were not analyzed here. In the following studies, we plan to focus on the deep mechanism about the ERS progress during IDD, especially the role of Ihh in the ERS, and the other crosstalk involving p-eIF2α/ATF4/Ihh signaling will become the principal research objective. Because so far there is less research on the role of Ihh in IDD, through our study, we believe Ihh can be used as an entry point to study the metabolism of NP cells in the process of IDD.

## Conclusion

**FIGURE 8:**
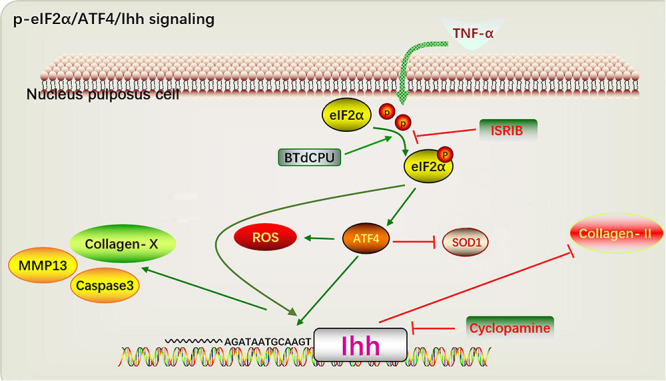
A proposed model depicting the p-eIF2α/ATF4/Ihh in regulating hypertrophy and apoptosis of NP cell. TNF-α and BTd promote the phosphorylation of eIF2α and upregulate the ATF4 expression, continuously resulting in the Ihh activation. Suppression of eIF2α phosphorylation by ISRIB decreases the ATF4 and Ihh levels and alleviates the hypertrophy and apoptosis of NP cell. Additionally, CPE treatment suppresses the hypertrophy and apoptosis of NP cell. Thus, disrupting the pathways ultimately protect against the development of IDD. p-eIF2α, phosphorylated eukaryotic initiation factor-2α; ATF4, activating transcription factor 4; Ihh, Indian hedgehog; NP, nucleus pulposus; TNF, tumor necrosis factor; BTd, BTdCPU; eIF2α, eukaryotic initiation factor-2α; CPE, cyclopamine.

Summarily, our study identified the promoting role of the p-eIF2α/ATF4/Ihh axis in the oxidative stress and apoptosis of NP cells. Inhibiting p-eIF2α was sufficient to protect the anti-oxidative enzyme, decrease the ROS, and suppress the apoptosis of NP cells. Besides, inhibiting Ihh was sufficient to suppress the apoptosis of NP cells. In general, pharmacological interruption of p-eIF2α/ATF4/Ihh protects NP cells (Figure 8). Further insights into the application of ISRIB or CPE might be a novel clinical treatment strategy for IDD.

## Data Availability Statement

The original contributions presented in the study are included in the article/supplementary material, further inquiries can be directed to the corresponding author/s.

## Ethics Statement

The studies involving human participants were reviewed and approved by the Ethics Committee of the Affiliated Zhongda Hospital of Southeast University. The patients/participants provided their written informed consent to participate in this study. The animal study was reviewed and approved by the animal experimental ethical inspection form of Southeast University. Written informed consent was obtained from the individual(s) for the publication of any potentially identifiable images or data included in this article.

## Author Contributions

XW and HC contributed to the study conception and design. XW, XH, and HC contributed to the funding support. JB, ZQ, and LL performed material preparation, all experiments, data collection and analysis. JB and HC provided valuable suggestions and help in the latest experiments for the revision. HC wrote the first draft of the manuscript. All authors commented on previous versions of the manuscript and approved the final manuscript.

## Conflict of Interest

The authors declare that the research was conducted in the absence of any commercial or financial relationships that could be construed as a potential conflict of interest.

## Footnotes

1 http://jaspar.genereg.net/

## Supplementary Material

Supplementary Figure 1 The surgical resection of mouse lumbar facet joints. **(A)** Exposed L3-4 level facet joints with reference to Jacoby’s line (yellow line). **(B)** After resection of the inferior articular process by microscissors. The yellow arrow indicates the supra supraspinous ligaments. **(C)** Mouse fur after surgery.

Supplementary Figure 2 The intervertebral disc height index (DHI) was calculated by 2 × (height of the posterior + middle + anterior parts of the disc)/(height of the posterior + middle + anterior parts of the two adjacent vertebral body).

Supplementary Figure 3 Representative magnetic resonance imaging (MRI) scans of patients with different categories of disc degeneration, according to Pfirrmann grade. G1 from the spine fracture, G3, G5 from the lumbar disc herniation. The yellow arrow indicates the surgical site.

Supplementary Figure 4 The efficiency of the siRNA transfection targeting ATF4. NP cells were transfected with the siATF4 or NC-siRNA, and the cells without transfection were set as control. **(A)** ATF4 protein levels were assessed by WB, measured by densitometric analyses and **(B)** expressed as folds relative to control. The siRNA transfection was over 80%. Data are presented as the means ± *SD* (*n* = 3) (^∗∗∗^*p* < 0.001).

Supplementary Figure 5 Upstream 2,000 bp section of the promoter region of the Ihh gene, and the putative DNA-binding sites of ATF4 protein (S1, S2).
