# Supplementary figures and images for: Pharmacological Disruption of Phosphorylated Eukaryotic Initiation Factor-2α/Activating Transcription Factor 4/Indian Hedgehog Protects Intervertebral Disc Degeneration via Reducing the Reactive Oxygen Species and Apoptosis of Nucleus Pulposus Cells
Source: Front Cell Dev Biol. 2021 Jun 7;9:675486. doi: 10.3389/fcell.2021.675486 (PMC8215438; doi:10.3389/fcell.2021.675486)

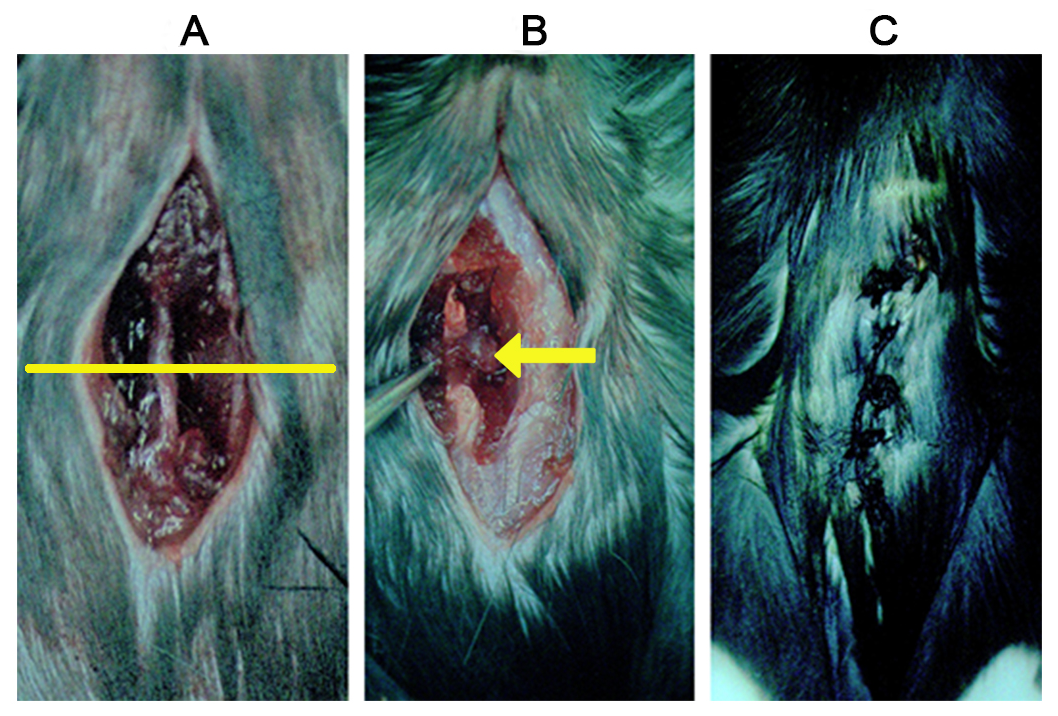

Supplement: Supplementary Figure 1 — The surgical resection of mouse lumbar facet joints. (A) Exposed L3-4 level facet joints with reference to Jacoby’s line (yellow line). (B) After resection of the inferior articular process by microscissors. The yellow arrow indicates the supra supraspinous ligaments. (C) Mouse fur after surgery. [file Image_1.TIF]

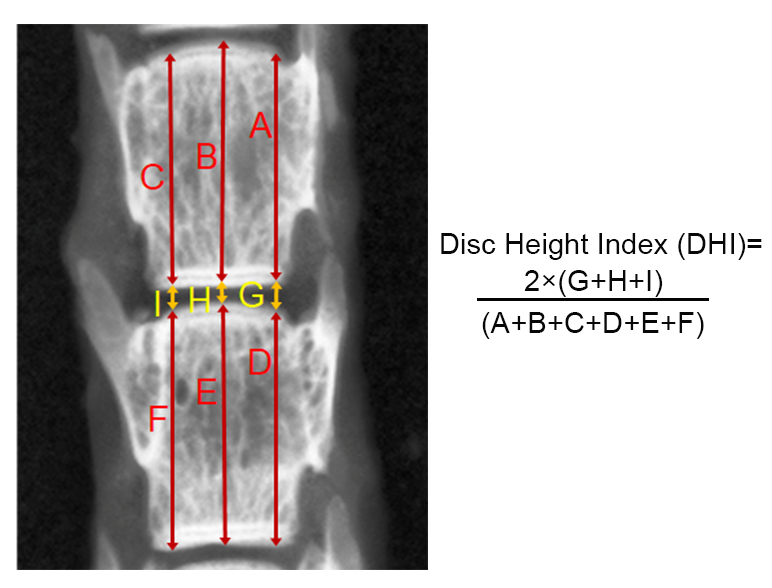

Supplement: Supplementary Figure 2 — The intervertebral disc height index (DHI) was calculated by 2 × (height of the posterior + middle + anterior parts of the disc)/(height of the posterior + middle + anterior parts of the two adjacent vertebral body). [file Image_2.TIF]

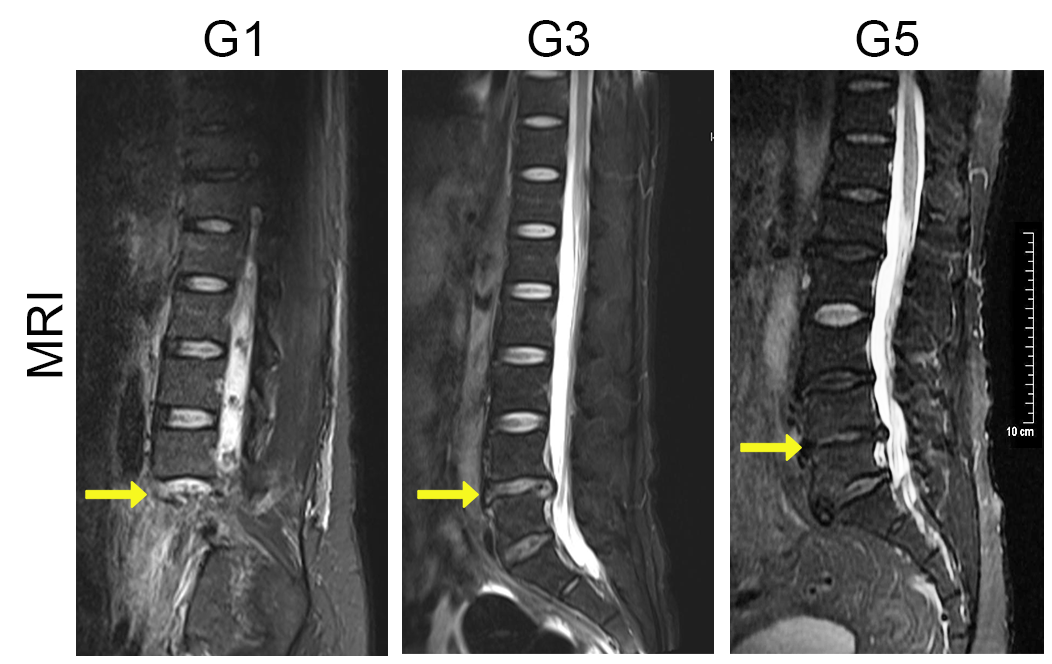

Supplement: Supplementary Figure 3 — Representative magnetic resonance imaging (MRI) scans of patients with different categories of disc degeneration, according to Pfirrmann grade. G1 from the spine fracture, G3, G5 from the lumbar disc herniation. The yellow arrow indicates the surgical site. [file Image_3.TIF]

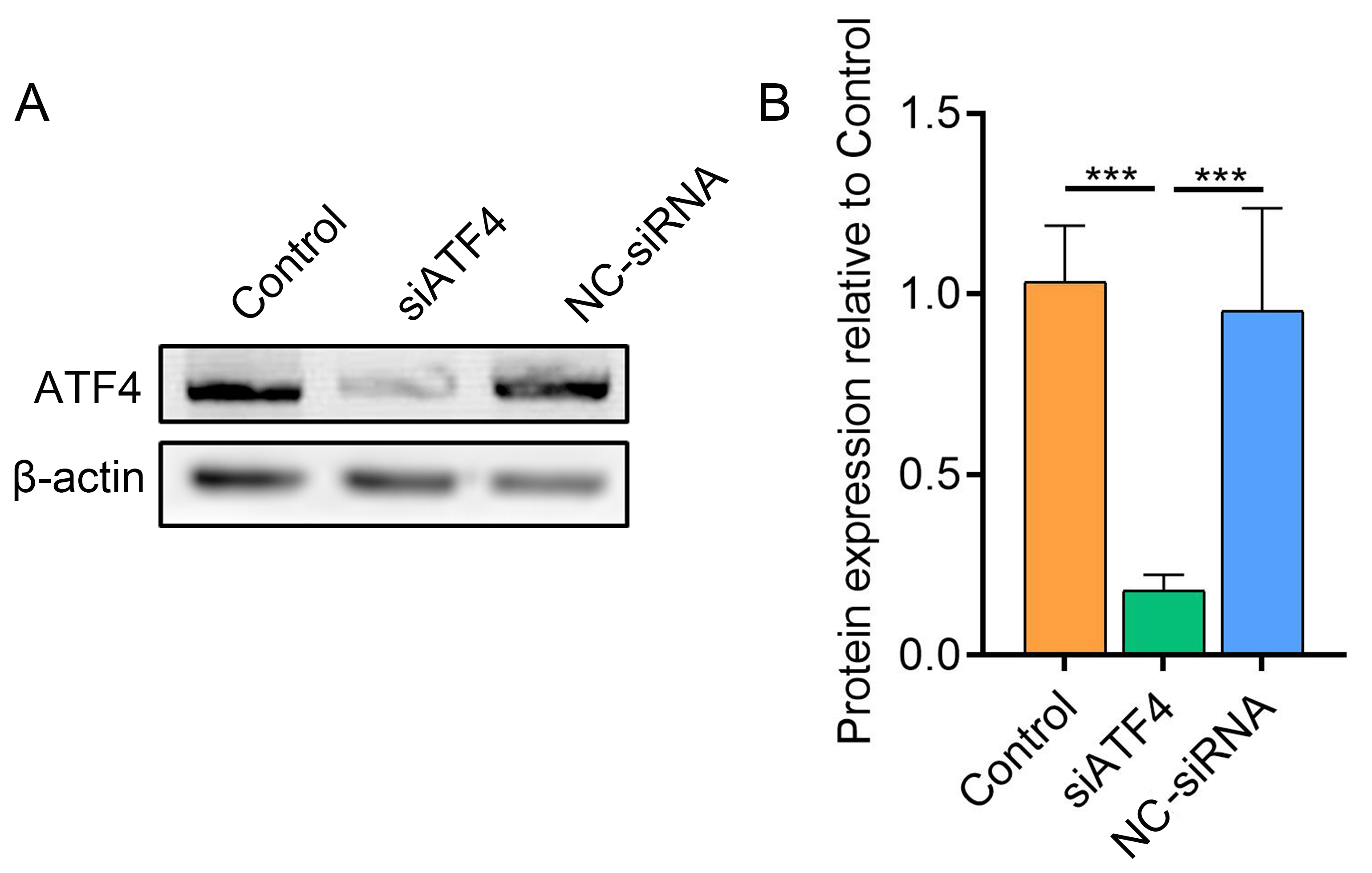

Supplement: Supplementary Figure 4 — The efficiency of the siRNA transfection targeting ATF4. NP cells were transfected with the siATF4 or NC-siRNA, and the cells without transfection were set as control. (A) ATF4 protein levels were assessed by WB, measured by densitometric analyses and (B) expressed as folds relative to control. The siRNA transfection was over 80%. Data are presented as the means ± SD (n = 3) (∗∗∗p < 0.001). [file Image_4.TIF]

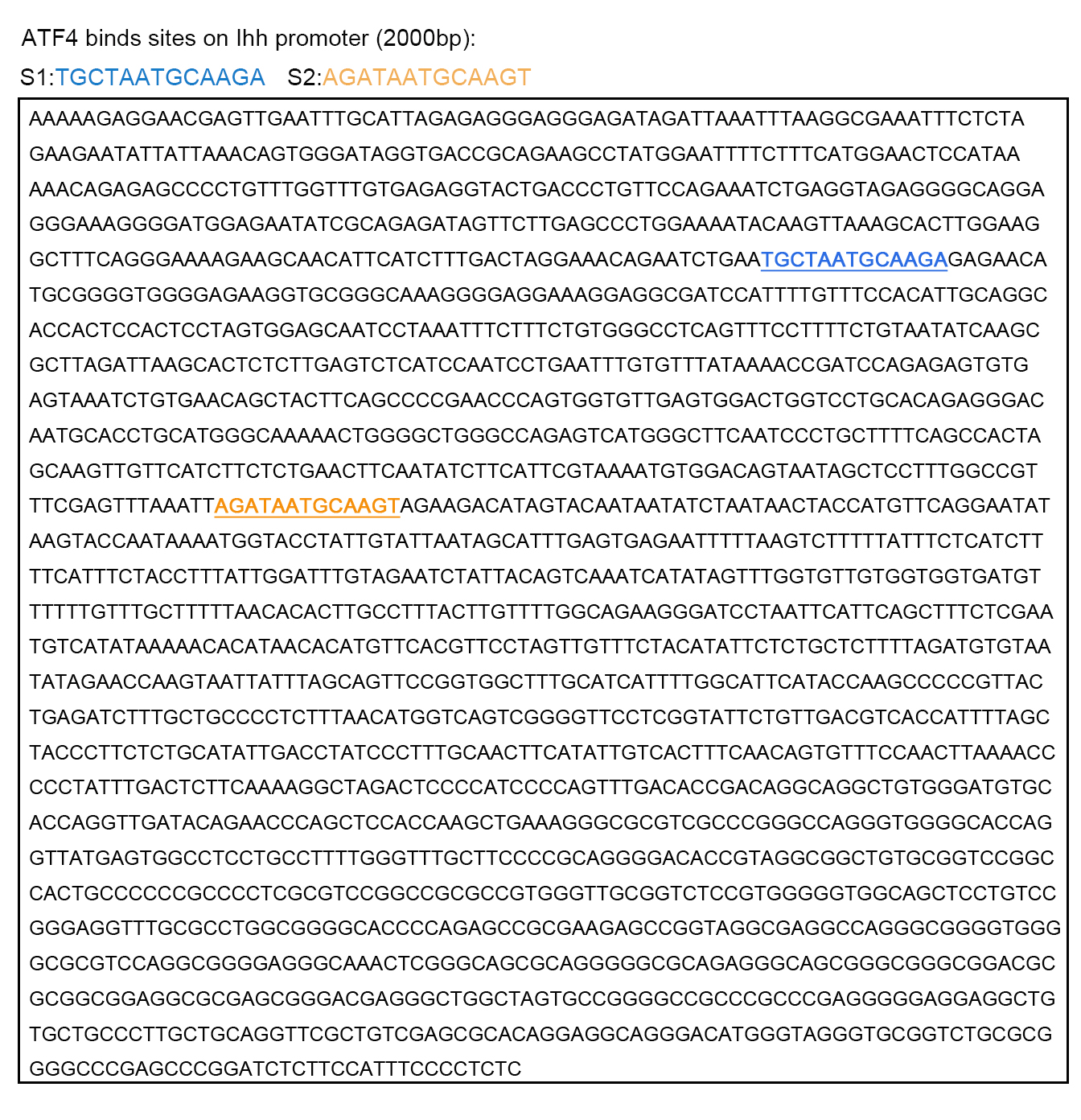

Supplement: Supplementary Figure 5 — Upstream 2,000 bp section of the promoter region of the Ihh gene, and the putative DNA-binding sites of ATF4 protein (S1, S2). [file Image_5.TIF]
