# Supplementary material for: Pharmacological Disruption of Phosphorylated Eukaryotic Initiation Factor-2α/Activating Transcription Factor 4/Indian Hedgehog Protects Intervertebral Disc Degeneration via Reducing the Reactive Oxygen Species and Apoptosis of Nucleus Pulposus Cells
Source: Front Cell Dev Biol. 2021 Jun 7;9:675486. doi: 10.3389/fcell.2021.675486 (PMC8215438; doi:10.3389/fcell.2021.675486)
Supplement: Supplementary file 6 [file Data_Sheet_1.docx]

**Supplementary Table.1 Primer sequences for PCR**

| **Binding site** | **Score** | **Primer Sequence** | **Length (bp)** |
| --- | --- | --- | --- |
| TGCTAATGCAAGA | 8.27 | 5`AGCCCTGGAAAATACAAGTT`3 | 188 |
|  |  | 3`GTAACGTCCGTGGTGAGGTG`5 |  |
| AGATAATGCAAGT | 7.89 | 5`TAAAATGTGGACAGTAATAG`3 | 103 |
|  |  | 3`ATTATTGATGGTACAAGTCC`5 |  |

**Supplementary Table.2 Patients information**

| **NO.** | **Gender** | **Age** | **Diagnosis** | **Grade** | **NO.** | **Gender** | **Age** | **Diagnosis** |  |
| --- | --- | --- | --- | --- | --- | --- | --- | --- | --- |
| 1 | F | 33 | LF | G1 | 13 | M | 52 | LDH | G3 |
| 2 | F | 41 | LF | G1 | 14 | M | 69 | LDH | G3 |
| 3 | M | 36 | LF | G1 | 15 | M | 57 | LDH | G3 |
| 4 | F | 43 | LF | G1 | 16 | F | 46 | LDH | G3 |
| 5 | M | 32 | LF | G1 | 17 | F | 49 | LDH | G5 |
| 6 | F | 44 | LF | G1 | 18 | F | 55 | LDH | G5 |
| 7 | M | 51 | LF | G1 | 19 | M | 64 | LDH | G5 |
| 8 | F | 37 | LF | G1 | 20 | F | 73 | LDH | G5 |
| 9 | M | 45 | LDH | G3 | 21 | M | 85 | LDH | G5 |
| 10 | F | 52 | LDH | G3 | 22 | M | 76 | LDH | G5 |
| 11 | F | 73 | LDH | G3 | 23 | F | 81 | LDH | G5 |
| 12 | F | 34 | LDH | G3 | 24 | M | 73 | LDH | G5 |
| NO. (Number); M (male); F (female); Lumbar fracture (LF); Lumbar disc herniation (LDH). | | | | | | | | | |
